# SEEING THROUGH A HOUSING LENS: STUDYING HOUSING-FOCUSED RESPONSES TO THE NEEDS OF OLDER ADULTS DURING THE PANDEMIC

**DOI:** 10.1093/geroni/igac059.1330

**Published:** 2022-12-20

**Authors:** Nancy Berlinger

**Affiliations:** The Hastings Center, Garrison, New York, United States

## Abstract

This paper draws on a study of unindexed literature produced by local and state policymakers, Area Agencies on Aging, nonprofit organizations, for-profit companies, age-friendly initiatives, affordable housing providers, and other groups responding to the needs of older adults at home during the COVID-19 pandemic. By collecting and analyzing descriptions of how service providers pivoted or improvised to meet urgent challenges, we sought to identify promising practices and policy ideas preserved in reports, newsletters, websites, and other communications. This paper reviews study methods and recommendations, and explores a concept that emerged from this research: recognizing services and support for older adults at home as “housing-focused” care that relies on the affordability, accessibility, and safety of a person’s home. The applications of the “housing lens” to future research on housing, aging, and health and to making progress on better integrating housing policy and health policy in aging societies will be discussed.

